# Diagnostic accuracy of smartphone technology for capturing videos and images of semen analysis samples: A cross-sectional study

**DOI:** 10.1080/20905998.2024.2441109

**Published:** 2024-12-23

**Authors:** Anju Khairwa, Pratibha Gautum, Richa Gupta

**Affiliations:** Departments of Pathology, University College of Medical Sciences & GTB Hospital, Delhi, India

**Keywords:** Smartphone, manual microscopy, semen analysis, diagnostic accuracy

## Abstract

**Background:**

Semen analysis is an essential indicator of male infertility potential. The study aims to assess the diagnostic accuracy of smartphones compared to manual microscopy for semen analysis.

**Method:**

It is a cross-sectional analytical study with investigator blinding. Data were collected from August to September 2023. Pictures of semen analysis were captured through light microscopy and stored in a coded format on a smartphone. Later, the results of both methods were compared.

**Results:**

A total of 50 adequate semen samples were included. The age of enrolled males was Mean±SD 29.4 ± 5.9 years. The sensitivity and specificity were 100% (95% CI 83.9%-100%) and (95% CI 88.1%-100%), respectively, for smartphones compared to light microscopy for total sperm counts. The positive predictive value (PPV) and negative predictive value (NPV) were both 100%, with (95% CI 83.9%-100%) and (95% CI 88.1%-100%), respectively. Sensitivity, specificity, PPV, and NPV for total sperm motility were 97.9%, 100%, 100%, and 66%, respectively. For normal morphology sperm, the sensitivity, specificity, PPV, and NPV of smartphones were 72.7%, 82.1%, 53.3%, and 91.4%, respectively, and for abnormal morphology sperm, they were 100%, 98%, 50%, and 100%, respectively. Smartphones exhibited a sensitivity of 98%, specificity of 100%, PPV of 100%, and NPV of 50% for assessing sperm vitality. The diagnostic agreement between smartphones and light microscopy was very good (κ value −0.6–1) for the detection of total count, vitality, and total motility of sperm.

**Conclusion:**

Smartphone technology demonstrates high sensitivity and specificity for semen analysis compared to manual microscopy. It also shows excellent agreement with manual microscopy for most parameters in semen analysis. We recommend smartphone reporting for semen analysis in remote areas and poor resource settings.

## Introduction

Semen analysis is an essential indicator of male infertility potential [1]. Worldwide, 8 to 12% of couples are estimated to be infertile [2]. In infertility cases, the male factor contributes 20–71% of the time [3,4]. Infertility often leads to depression, helplessness, frustration, and wearing down of infertile couples [5]. Zargar et al. reported that approximately 50% of male infertility cases are due to abnormalities of the reproductive tract, with the etiology being unknown in 25% of cases [6]. To determine the etiology of male infertility, semen analysis plays a crucial role. Semen analysis typically involves counts and sperm motility measurements using standardized methods. The World Health Organization (WHO) has published a series of manuals for the standardized laboratory examination and processing of semen analysis [1,7,8]. The aim of these WHO manuals is to establish a uniform reporting system worldwide and standardize the procedure and various basic semen tests [7,8]. The latest edition, the 6th edition published in 2021, emphasizes reference ranges, introduces new terminology, assists in infertility and fertility diagnostic tests, and addresses the management of male reproductive health [1].

The most commonly used method for semen analysis is direct manual light microscopy. However, various technologies, such as automated semen analysis, are now being utilized [9]. Additionally, smartphones are emerging as a potential tool for semen analysis. Smartphones are already being used in different clinical settings, including self-care for diabetes, point-of-care nucleic acid diagnosis, and computer-assisted semen analysis [10–13].

### Rationale/Justification for the conduct of this study

Semen analysis requires fresh samples and immediate analysis to prevent spoilage and reduce sperm motility and viability. In remote areas where peripheral laboratories are situated, such as primary health care centers (PHCs- primary medical care where patients met health workers initially)) and community health centers (CHCs- offer people in the medically uninsured, underinsured & lesser income groups with primary and preventive care), there may be a lack of doctors or only junior doctors available, especially during emergency duties. In such situations, semen examination can be conducted using a smartphone by capturing pictures and videos to be sent for reporting to an expert. In areas where doctors are not readily available, technical staff can send pictures of the microscope to a doctor for an opinion or to report the sample. With these considerations in mind, our study aims to assess the diagnostic accuracy of Smartphone technology for capturing videos and images of semen analysis samples compared to manual microscopy for semen analysis. Our primary objectives are to estimate the sensitivity, specificity, positive predictive value (PPV), and negative predictive value (NPV) of Smartphones for semen analysis compared to manual microscopy.

## Method and materials

### Study design

The study design is a cross-sectional analytical study with investigator blinding. The study was performed in the tertiary care center’s fluid section of the Department of Pathology, with data collected prospectively from August to September 2023.

### Sample size

Since there was no similar study to calculate the sample size, we selected a convenient sample size of 50.

### Inclusion criteria

All adequate semen samples (as per WHO 2021 guidelines, semen sample volume of 1.4 ml (1.3–1.5 ml) were considered adequate) [15] received in the fluid section during the study period.

### Exclusion criteria

Inadequate samples with a volume of <1.3 ml (as per WHO 2021 guidelines, semen sample volume of 1.3 to 1.5 ml considered adequate) [15] or contaminated samples with urine or other substances were excluded.

### Procedure and processes

In this study, we compared the reporting of two diagnostic techniques: manual light microscopy and pictures & videos captured by Smartphones for semen analysis. Informed consent was obtained from all participants. Semen analysis was conducted according to the WHO 2021 guidelines/manual [15]. Outpatient department (OPD) samples received for routine microscopic analysis of semen were reported as routine through light microscopy. Additionally, these samples were examined under light microscopy, and pictures (21 per case) and one video were captured of semen slides and Neubauer hemacytometer using a Smartphone by a senior resident (PG) doctor and stored in a coded format on the phone. Pictures and videos were captured by manually holding the Smartphone camera on the eyepieces of the microscope without connecting any device.

**Figure 1.**
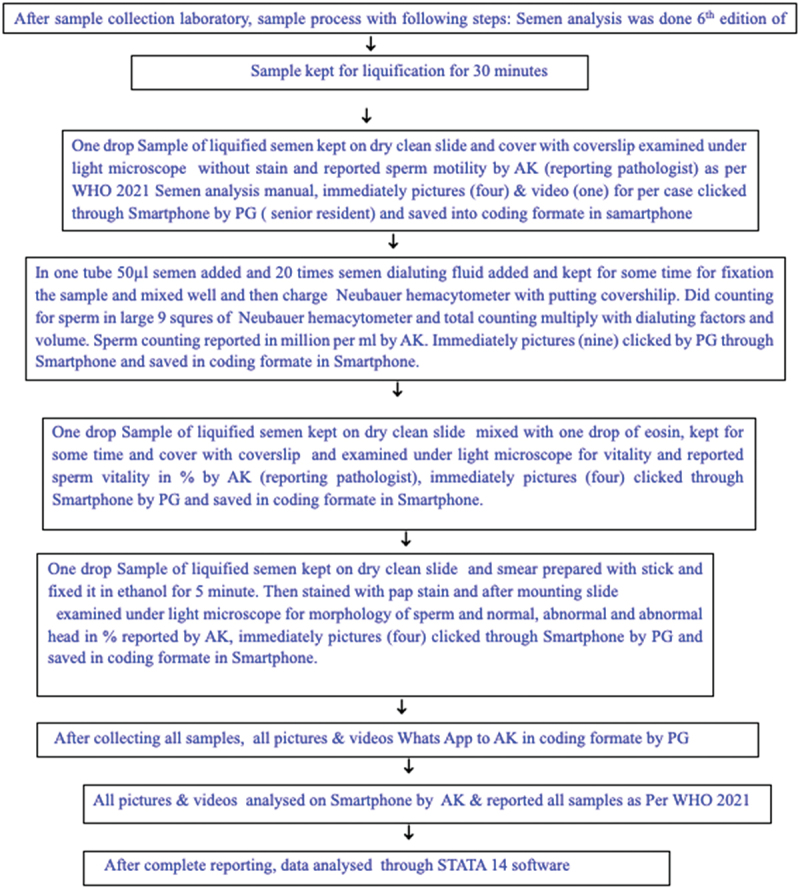
Flowchart for processing of semen analysis.

Subsequently, the pictures and videos were sent via WhatsApp to the reporting pathologist (AK) on a smartphone. The reporting pathologist, who initially reported using manual light microscopy, also reported the pictures and videos captured on the Smartphone but was blinded to the coding format. All pictures and video recordings were taken on fresh semen samples. Semen sample analysis included motility assessment in direct smears without stain (four pictures were taken [through- one microscopic field divided into four corners by an imaginary line] and one video recorded), viability assessment in eosin-stained smears (four pictures), and morphological assessment on Pap-stained smears (four pictures). Sperm counting was performed in a large 9-square Neubauer hemacytometer without stain. The hemacytometer was charged with diluted semen at a ratio of 1:20 with diluting fluid. Semen analysis was conducted in accordance with the latest WHO 2021 guidelines,15 with total sperm count reported in million/ml and motility calculated as a percentage (progressive motile – PM, non-progressive motile – NPM, non-motile – NM). Viability was calculated based on eosin staining (viable sperm in %, dead sperm in %, and retained stain due to pore presence). Morphology was assessed for normal sperm %, abnormal sperm %, and head abnormality. Semen analysis procedure shown in Figure 1 as flow chart.

### Statistical analysis

We conducted statistical analysis using STATA 14 software. Continuous data were presented as mean ± standard deviation (SD) for normally distributed variables and median with an interquartile range (IQR) for skewed variables. Categorical data were reported as percentages. The sensitivity, specificity, positive predictive value (PPV), negative predictive value (NPV), and accuracy of smartphones were calculated against direct microscopy (manual), which was considered the gold standard. Cohen’s Kappa statistics (linear κ) were used to assess the agreement between smartphone and light microscopy reporting. The quality of agreement was defined as follows: κ-value (<0.20) indicating poor agreement, κ-value (0.21–0.40) indicating fair agreement, κ-value (0.41–0.60) indicating moderate agreement, κ-value (0.61–0.80) indicating good agreement, and κ-value (0.81–1.0) indicating very good agreement between the two testing methods. A p-value of < 0.05 was considered statistically significant.

## Results

A total of 50 samples were collected over a two-month period. The mean age of enrolled males was 29.4 ± 5.9 years. The mean volume of semen was 1.8 ± 0.4, and the liquefaction time was 30 ± 0 minutes for all semen samples. The color of all semen specimens was grey-white. Table 1 presents all semen analysis parameters with their respective values.

**Table 1.** Different parameters of semen analysis with exact values are demonstrated.

S.N.	Parameters	Reporting on Light Microscope*	Reporting on Smartphone*
1	Total count of Sperm million/ml	25 (8.5, 70)	23 (9, 69)
2	Total Motility (%)	60 (50, 70)	60 (50, 66)
3	PM (%)	50 (45, 65)	50 (45, 60)
4	NPM (%)	5 (5, 10)	5 (5, 10)
5	IM (%)	40 (30, 45)	40 (35, 50)
6	Vitality (%)	70 (65, 80)	80 (70, 85)
7	NM (%)	80 (75, 85)	80 (75, 85)
8	AM (%)	20 (20, 25)	20 (15, 25)
9	AHM (%)	15 (10, 15)	15 (10, 15)

*Median (IQR) values, PM- Progressive motility, NPM- Non-progressive motility, IM- Immotile, NM- Normal morphology, AM- Abnormal morphology, AHM- Abnormal Head morphology.

This table presents the median (with interquartile range) values for each parameter reported on both the light microscope and smartphone.

The sensitivity is 100% with a 95% confidence interval (CI) of (83.9%-100%), and the specificity is 100% with a CI of (88.1%-100%) for Smartphone technology (captured pics & video used to report semen) for total counts of sperm million/ml in semen analysis compared to light microscopy. The positive predictive value (PPV) is 100% with a 95% CI of (83.9%-100%), and the negative predictive value (NPV) is 100% with a 95% CI of (88.1%-100%) for total sperm count in semen analysis compared to light microscopy. Table 2 depicts the sensitivity, specificity, PPV, and NPV of smartphone reporting for the detection of sperm motility compared to light microscopy reporting.

**Table 2.** Sensitivity and specificity of smartphone compared to light microscopy for detection of sperm motility in semen analysis.

Different Types of Motility	Statistical Parameters							
	Sensitivity (%)	95% CI	Specificity (%)	95% CI	PPV (%)	95% CI	NPV (%)	95% CI
Total Motility	97.9	(88.9–99.9)	100	(15.8–100.0)	100	(92.5–100)	66	(9.4–99.2)
PM	100	(92.1–100.0)	100	(47.8–100.0)	100	(92.1–100.0)	100	(47.8–100.0)
NPM	88.9	(51.8–99.7)	97.6	(87.1–99.9)	88.9	(51.8–99.7)	97.6	(87.1–99.9)
IM	100	(39.8–100)	100	(92.3–100)	100	(39.8–100)	100	(92.3–100)

PM- Progressive motility, NPM- Non-progressive motility, IM-Immotile, 95% CI (confidence interval).

Smartphone technology demonstrates very good sensitivity, specificity, positive predictive value (PPV), and negative predictive value (NPV) for detecting all types of sperm motility in semen analysis, as shown in Table 2.

In addition, Smartphones exhibit high accuracy in detecting sperm vitality in semen analysis, with a sensitivity of 98% and a 95% confidence interval (CI) of (89.1–99.9), specificity of 100% with a 95% CI of (2.5–100%), PPV of 100% with a 95% CI of (92.6–100), and NPV of 50% with a 95% CI of (1.3–98.7). The diagnostic accuracy of Smartphone for detecting semen morphology parameters is detailed in Table 3.

**Table 3.** Sensitivity, specificity, PPV, and NPV of smartphone compared to light microscopy for detection of sperm morphology in semen analysis.

Different Parameters of Sperm Morphology	Statistical Parameters							
	Sensitivity (%)	95% CI	Specificity (%)	95% CI	PPV (%)	95% CI	NPV (%)	95% CI
NM	72.7	(39.0–94.0)	82.1	(66.5–92.5)	53.3	(26.6–78.7)	91.4	(76.9–98.2)
AM	100	(2.5–100.0)	98	(89.1–99.9)	50	(1.3–98.7)	100	(92.6–100.0)
AHM	100	(2.5–100.0)	98	(89.1–99.9)	50	(1.3–98.7)	100	(92.6–100.0)

NM- Normal morphology, AM- Abnormal morphology, AHM-, Abnormal Head morphology, 95% CI (confidence interval), PPV-positive predictive value, NPV-Negative predictive value.

Further, we estimated the diagnostic agreement between light microscopic reporting and Smartphone reporting using Cohen’s Kappa statistics (linear κ). The diagnostic agreement was very good (κ value of 1) and 100% for detecting the total sperm count in million/ml. The agreement for different types of sperm motility is depicted in Table 4.

**Table 4.** Diagnostic agreement between smartphone reporting and light microscopy reporting for different types of sperm motility in semen analysis.

Different Types of Sperm Motility	Agreement (%)	Linear κ	Standard Error	95% CI
Total Motility	98.0	0.7899	0.1383	0.1383
Progressive Motile	100.0	1.000	0.1414	0.1414
Non-Progressive Motile	96.0	0.8645	0.1414	0.1414
Non-Motile	100.0	1.000	0.1414	0.1414

**Figure 2.**
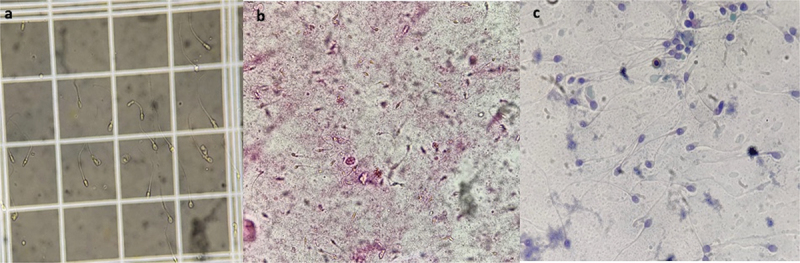
Depicting different parameters of semen analysis by smartphone (a) sperm counting million/ml through neubarchamber, (200x) (b) sperm vitality, (eosin stain, 200X) (c) sperm morphology, (pap stain, 1000x, Video recording for sperm motility, (100x).

The Smartphone technique demonstrates very good agreement with light microscopy for detecting sperm motility. The agreement for the detection of sperm vitality was good (98% with κ = 0.6575) for Smartphone semen analysis compared to light microscopy. The diagnostic agreement of Smartphone for the detection of sperm morphology compared to light microscopy is shown in Table 5. Figure 2 Depicts different parameters of semen analysis through Smartphone: (a) Sperm counting in million/ml, (b) Sperm vitality, (c) Sperm morphology, and Video 1 recording for Sperm Motility.

**Figure 3.**
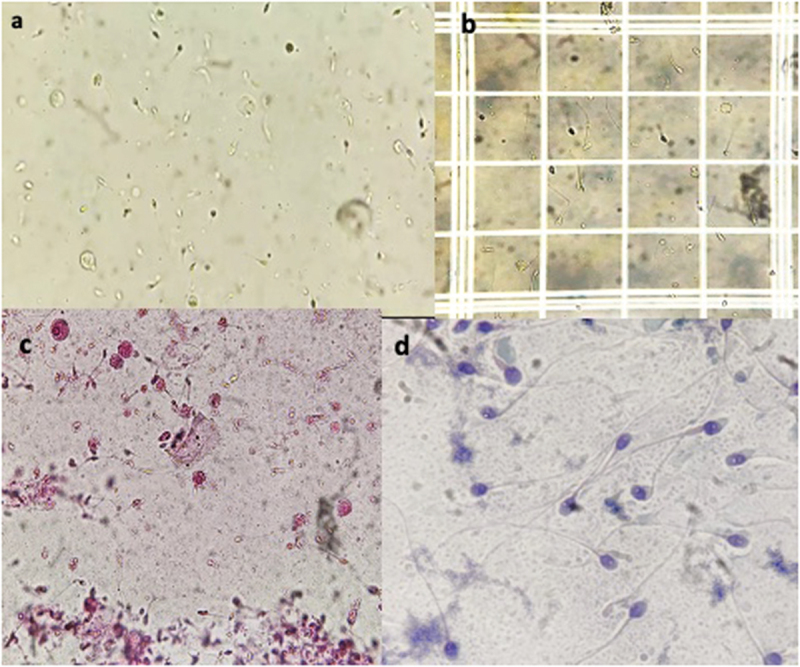
Panel of microprograms captured by smartphone, (a) for sperm motility on unstained slide (x200); (b) sperm counting million/ml through neubarchamber (100x); sperm vitality, (eosin stain, x200) (c) sperm morphology, (pap stain, 1000x, Video recording for sperm motility, (100x).

Figure 3 Shows additional pictures captured on the Smartphone through a light microscope for semen analysis.

Figure 3 Shows additional pictures captured on the Smartphone through a light microscope for semen analysis

**Table 5.** Diagnostic agreement for detection of sperm morphology in semen analysis between smartphone and light microscopy.

Different Parameters of Sperm Morphology	Agreement (%)	Linear κ	Standard Error	95% CI
Normal Morphology	50.0	0.3344	0.0764	0.0764
Abnormal Morphology	50.0	0.3344	0.0764	0.0764
Abnormal Head Morphology	58.0	0.3906	0.0849	0.0849

## Discussion

In the present study, we observed very good diagnostic accuracy and cost benefits of smartphones (captured pictures and videos used for reporting semen) in semen analysis, especially for most parameters, compared to light microscopy reporting. Smartphone technology exhibited 100% sensitivity and specificity, along with 100% PPV and NPV for detecting the total sperm count. Smartphone-based analysis also demonstrated high sensitivity (ranging from 97.9% to 100%), specificity (ranging from 97.6% to 100%), PPV (ranging from 88.9% to 100%), and NPV (ranging from 66% to 100%) for sperm motility detection. Furthermore, for sperm vitality detection, Smartphones exhibited 98% sensitivity and 100% specificity. Moreover, Smartphones showed very good sensitivity (ranging from 97% to 99%) and specificity (ranging from 82.1% to 98%) for detecting normal and abnormal sperm morphology. Notably, Smartphones demonstrated high sensitivity (100%) and specificity (98%) for the detection of abnormal sperm morphology, particularly head abnormality. This high accuracy is attributed to the ability to zoom in on the pictures and videos captured by Smartphones, enabling detailed analysis. Results such as these are seldom reported in the literature.

However, a few studies have explored semen analysis using Smartphone assistance [11,12]. Cheon et al. conducted a study on Smartphone-based computer-assisted semen analysis (CASA) compared with manual microscopy semen analysis and found it to be a cost-effective approach, motivating clinic visits for early diagnosis and treatment [12]. They also claimed to follow-up the patients and develop new software for morphology analysis [12]. Our study, employing a different method, is more feasible and cost-effective, requiring no additional assistance techniques, thus making it suitable for resource-poor countries.

Another study by Tsao et al. reported point-of-care semen analysis using Smartphones and colorimetric paper-based diagnostic devices, offering an inexpensive and equipment-free evaluation process with high accuracy [13]. They found sensitivity was 96%, specificity was 65%, PPV was 75%, NPV was 92.9%, and accuracy was 80.9% with Smartphone recording and analytic system, whereas using visual observation methods, sensitivity 41%, specificity 95%, PPV 90%, NPV 59.4%, and accuracy was 67% [13]. However, our study demonstrated higher sensitivity, specificity, and diagnostic accuracy of Smartphones compared to visual observation methods. Moreover, our study design is more cost-effective as it does not require additional assistance techniques and does not necessitate expertise in the technique.

Comparatively, recent studies have described automated semen analysis using various techniques, including SQA® Vision, as a reliable and high-throughput method [9,16]. However, these studies reported poor agreement for some parameters between manual and automated semen analysis [9,16]. In contrast, our study demonstrated 100% agreement with Smartphones in progressive motility and very strong (k value-1) agreement for other parameters of semen analysis. Smartphone technology captures images and videos at a low cost for reporting semen, making it a favourable option over automated semen analysers, which are costly and time-consuming.

Park et al. reported a sensitivity of 92.6%, specificity of 66.7%, and overall accuracy rate of 84.6% for smartphone-based app-assisted semen analysis by O’VIEW-M SQA® [17]. However, this technique exhibited lower diagnostic accuracy compared to our study and was both costly and time-consuming. In contrast, Smartphone technology in our study proved to be a cost-effective and rapid method for semen analysis compared to sample transport from remote areas. Such comparisons are rarely found in the literature, and we recommend the implementation of such inexpensive technology in routine practice. Semen analysis using Smartphone-captured images and videos may facilitate sample reporting from peripheral centers by experts in the field and could also serve as a valuable tool for training purposes.

Limitations of our study include the 1. Lack of comparison with other studies, 2. a lack of patient follow-up correlation, 3. single-center design, and 4. inability to compare with cytogenetics.

## Conclusion

Our study introduces a novel diagnostic approach to semen analysis through Smartphone technology, utilizing captured images and videos. It is highly accurate, cost-effective, and rapid compared to light microscopy analysis of semen samples, particularly from remote areas. Smartphone technology presents a valuable tool for semen sample analysis in resource-poor countries and remote areas.
